# Tissue oxygen saturation as an early indicator of delayed lactate clearance after cardiac surgery: a prospective observational study

**DOI:** 10.1186/s12871-015-0140-7

**Published:** 2015-10-30

**Authors:** Rüdger Kopp, Katja Dommann, Rolf Rossaint, Gereon Schälte, Oliver Grottke, Jan Spillner, Steffen Rex, Gernot Marx

**Affiliations:** Department of Intensive Care, University Hospital RWTH Aachen, RWTH Aachen University, Pauwelsstr. 30, 52074 Aachen, Germany; Department of Anaesthesiology, University Hospital RWTH Aachen, RWTH Aachen University, Pauwelsstr. 30, 52074 Aachen, Germany; Department of Thoracic and Cardiovascular Surgery, University Hospital RWTH Aachen, RWTH Aachen University, Pauwelsstr. 30, 52074 Aachen, Germany; Department of Anaesthesiology, University Hospitals Leuven, Herestraat 49, 3000 Leuven, Belgium; Department of Cardiovascular Sciences, Katholieke Universiteit Leuven, Herestraat 49, 3000 Leuven, Belgium

**Keywords:** Cardiac Surgical Procedure, Microcirculation, Lactic acid, Haemodynamics, Tissue oxygenation

## Abstract

**Background:**

In this observational study near infrared spectroscopy (NIRS) was evaluated as a non-invasive monitor of impaired tissue oxygenation (StO_2_) after cardiac surgery. StO_2_, cardiac output, mixed venous oxygen saturation and mean arterial pressure were compared with lactate clearance as established measure for sufficient tissue perfusion and oxygen metabolism.

**Methods:**

Forty patients after cardiac surgery (24 aortocoronary bypass grafting, 5 heart valve, 3 ascending aorta and 8 combined procedures) were monitored until postoperative day 1 with NIRS of the thenar muscle (InSpectra™ StO_2_-monitor, Hutchinson Technology), a pulmonary-artery catheter and intermittent blood gas analyses for the assessment of lactate clearance.

**Results:**

StO_2_ was reduced 4 h after surgery (75 ± 6 %), but recovered at day 1 (84 ± 5 %), while lactate concentration remained increased. Using uni- and multivariate regression analysis, minimum StO_2_ (*r* = 0.46, *p <0.01*) and cardiac index (*r* = 0.40, *p <0.05*) correlated with lactate clearance at day 1, while minimum mixed venous saturation and mean arterial pressure did not. In a receiver-operating characteristics (ROC) analysis, minimum StO_2_ (with a threshold of 75 %) predicted a lactate clearance <10 % at day 1 with an area under the ROC-curve of 0.83, a sensitivity of 78 % and a specificity of 88 %. In the subgroup with StO_2_ *<*75 %, troponin and creatine kinase MB were significantly increased at day 1.

**Conclusions:**

StO_2_ below 75 % in the first hours after surgery was a better early indicator of persistent impaired lactate clearance at day 1 than cardiac index, mixed venous oxygen saturation or mean arterial pressure.

## Background

After cardiac surgery, haemodynamics and systemic oxygenation are often impaired and clinical guidelines address sufficient tissue perfusion and oxygen metabolism as primary goal of haemodynamic management [1]. Recommended monitoring of therapeutic goals includes direct and indirect parameters of global haemodynamics, such as cardiac index, arterial and central venous blood pressure, as well as central (S_cv_O_2_) or mixed venous oxygen saturation (S_v_O_2_). Additionally lactate concentration should be measured as a surrogate parameter of tissue hypoxia. In a prospective observational study immediate as well as delayed hyperlactatemia was associated with increased morbidity and mortality after 325 cardiopulmonary bypass operations [2]. Lindsay et al. demonstrated in a retrospective study that lactate concentration and clearance were associated with mortality, reoperation and morbidity of 1291 patients after cardiac surgery [3].

To overcome some limitations of indirectly monitored tissue perfusion and oxygenation near-infrared spectroscopy (NIRS) was developed measuring directly and continuously tissue oxygen saturation (StO_2_) [4, 5]. In critically ill patients evaluation of StO_2_ demonstrated for example, that minimum StO_2_ predicted multiple organ failure after severe trauma patients [6] and after early goal-directed therapy of septic shock, a StO_2_ value below 78 % was associated with increased mortality at day 28 [7].

Even after major non-cardiac surgery, minimum intraoperative StO_2_, but not mean StO_2_, was associated with a composite endpoint of major postoperative complications and mortality in a prospective observational study [8]. For cardiac surgery Uilkema and Groeneveld could demonstrate, that StO_2_ decreased significantly up to 22 h after surgery [9]. In 74 adults undergoing cardiac surgery, perioperative reduced StO_2_ was associated with increased postoperative morbidity 3 and 15 days after surgery [10].

The aim of this study was to evaluate the potential of near-infrared spectroscopy as a direct and early indicator of impaired tissue oxygenation after cardiac surgery. We tested the hypothesis, that StO_2_ in the first hours after cardiac surgery could predict a late hyperlactatemia and delayed lactate clearance on the first postoperative day.

## Methods

In this prospective observational study, StO_2_ was measured in 40 patients with elective cardiac surgery using non-invasive StO_2_-monitoring (InSpectra™ StO_2_, Hutchinson Technology Inc., Hutchinson, Minnesota, USA) in the period from January 2008 to June 2008. The study was approved by the RWTH Aachen University regional research ethics committee (Ref: EK 161/07) and met the Declaration of Helsinki criteria. The patients were included the day before surgery by chance, when written informed consent could be obtained for participation in the study including data publication. The reporting of this study follows the STROBE statement for observational studies [11]. Patient number was assumed from already published observational studies on StO_2_ [12–14]. Inclusion criteria were surgery for aortocoronary bypass, valve repair or replacement of the ascending aorta as well as a combination of these operations. Exclusion criteria were pregnancy, age younger than 18 years, emergency operation, a moribund status before operation or a contraindication for extended haemodynamic monitoring with a pulmonary artery catheter (PAC). EURO score, age, sex and type of operation were recorded.

### Anaesthesia

Anaesthesia, haemodynamics, fluid management and blood transfusion were managed according to in-house standard. Patients received an oral premedication with 7.5 mg midazolam and anaesthesia was induced with etomidate 0.1 mg · kg^−1^, sufentanil 0.5-1.0 μg · kg^−1^ and rocuronium 1 mg · kg^−1^. Anaesthesia was maintained with sufentanil 1.0 μg · kg^−1^ · hr^−1^, isoflurane (0.4–1.0 minimum alveolar concentration) and propofol 2-4 mg kg^−1^ · hr^−1^ during cardiopulmonary bypass. Installation of the patients included orotracheal intubation, arterial and central venous catheterization, placement of a PAC via an introducer sheath in the jugular vein, and transurethral catheterization. Parameters of systemic haemodynamics including cardiac index, lactate concentration, base deficit and S_v_O_2_ were measured. The StO_2_ sensor was placed on the thenar muscle of the right hand in a position, which ensured an adequate total haemoglobin index (THI) of more than 5.0 according to the recommendation of the manufacturer. A system check was performed before application of the sensor. At the different time points, a THI greater than 5.0 was confirmed before recording of StO_2_ to avoid artefacts. To measure tissue oxygen saturation near-infrared light (680–800 nm) is emitted into the thenar tissue with a depth of about 15 mm. In the light that is reflected from the tissue, the percentage of oxygenated haemoglobin is calculated by using the different absorption spectra of oxygenated and deoxygenated haemoglobin.

### Intensive Care Unit

At end of surgery, patients were transferred to the Intensive Care Unit (ICU) and analgosedation was maintained with sufentanil 0.5-1.0 μg · kg^−1^ · hr^−1^ and propofol 1-3 mg · kg^−1^ · hr^−1^. Haemodynamics were stabilized with intravenous fluid administration, noradrenaline as vasopressor and adrenaline for inotropic support depending on S_v_O_2_, cardiac output, blood pressure and clinical assessment according to clinical guidelines [1]. After respiratory and haemodynamic stability as well as normothermia had been achieved, analgosedation was terminated to extubate patients.

### Data acquisition

Complete data were captured after induction of anaesthesia (T −1), at the end of surgery (T 0), 1 h (T 1), 4 h (T 4) and the next morning after surgery (T d1). At these time points pulmonary capillary wedge pressure, cardiac index and S_v_O_2_ were measured and actual values of continuously measured haemodynamic parameters (StO_2_, heart rate, arterial, pulmonary arterial, central venous pressure) were prospective recorded. In addition, arterial blood gas analyses were performed for the assessment of lactate concentration, base deficit, pH, haemoglobin concentration and oxygen saturation (S_a_O_2_). Lactate clearance was calculated as previously published [15]: (lactate_T0_–lactate_Tx_) · lactate_T0_^−1^ · 100 %.

Laboratory values, fluid balance and application of vasoactive drugs were recorded according to local standard. Aortic cross-clamping time, cardiopulmonary bypass time, length of ICU stay, duration of mechanical ventilation and SOFA score were registered from patient data.

### Statistical analysis

Mean and standard deviation (SD) were calculated. After confirmation of normal distribution with the Kolmogorov-Smirnov test, differences between time points were tested using ANOVA. When repeated measures ANOVA detected statistical significance, it was followed by a Tukey multiple comparisons post-test for dependent samples to correct for multiple measurements. Statistical significance between subgroups was tested with unpaired t test after confirmation of normal distribution.

Univariate and multiple regression analysis were performed to assess the correlation between cardiac index (CI), S_v_O_2_, mean arterial pressure (MAP) and lactate clearance. InStat Statistical Software version 3.06 (GraphPad, San Diego, USA) was used for statistical analysis and a *p-value <0.05* was considered significant. Sigma Plot 11.0 Software (Systat Software, Inc., San José, USA) was used for the assessment of receiver operating characteristic (ROC) curves and the definition of an optimum threshold by calculating the maximum Youden index (J = Sensitivity + Specifity-1).

## Results

In this observational study, 40 patients after cardiac surgery (30 male and 10 female) were included. Complete data including outcome were received from all patients. Demographic data are presented in Table 1 including mean Euro-Score and type of operation.

**Table 1:** Base line data of patients

		Mean (SD)	Median	Range
Age	years	68.7 (8.0)	70	45–85
Male	n	30		
Female		10		
Euroscore	value	6 (4)	7	0–12
	%	10.4 (10.2)	6.1	0.9–35.2
Type of operation		Number	%	
Isolated Aortocoronary bypass	n	24	60.0	
off pump		7	17.5	
Combined aortocoronary bypass	n	7	17.5	
Other (including combination)	n	16	40.0	
Aortic valve		8	20.0	
Mitral valve		3	7.5	
Aortic and mitral valve		2	5.0	
Ascending aorta		3	7.5	
Cardiopulmonary bypass	n	33	82.5	

After ICU admission, StO_2_ was significantly reduced (T 1 and T 4) and recovered at day 1 (T d1), whereas other parameters of tissue oxygen delivery and consumption including lactate concentration and base deficit demonstrated significantly higher values at T d1 compared to T −1 (Table 2). After surgery, cardiac index declined at T 1 and T 4 followed by a recovery at T d1. S_v_O_2_ was persistently reduced after T −1. Table 2 summarizes haemodynamic data and the results of the blood gas analysis.

**Table 2:** Haemodynamics, gas exchange and metabolic state of patients

	T −1	T 0	T 1	T 4	T d1
	After induction of anaesthesia	After surgery	1 hour after surgery	4 hours after surgery	Day 1
StO_2_ (%)	85 (4)*	86 (6)**	76 (7)*^,^**^,^***	75 (6)*^,^**^,^****	84 (5)***^,^****
THI	13.5 (1.8)*	13.5 (2.0)**	12.6 (1.6)*^,^**	12.4 (1.5)*^,^**	12.6 (2.1)*^,^**
Heart rate (min^−1^)	64 (12)*	87 (9)*	88 (8)*	91 (8)*	91 (9)*
MAP (mmHg)	84 (15)	80 (12)	81 (12)	77 (11)	82 (15)
MPAP (mmHg)	24 (8)	25 (11)	26 (6)***	27 (7)****	22 (7)***^,^****
CVP (mmHg)	12 (4)*	12 (5)**	12 (3)	12 (4)****	9 (4)*^,^**^,^****
PCWP (mmHg)	16 (6)*	13 (5)**	11 (4)*^,^**^,^***	11 (4)*^,^**^,^****	8 (5)*^,^**^,^***^,^****
CI (L min^−1^ m^−2^)	2.0 (0.4)*	2.5 (0.7)*^,^**	2.1 (0.7)**^,^***	2.4 (0.6)*	2.7 (0.8)*^,^***
Body temperature (°C)	36.0 (0.5)*	36.0 (0.8)**	35.4 (0.9)*^,^**^,^***	36.3 (0.9)***^,^****	37.2 (0.5)*^,^**^,^***^,^****
FiO_2_	0.9 (0.2)*	1.0 (0.0)**	0.6 (0.1)*^,^**^,^***	0.4 (0.1)*^,^**^,^***	0.3 (0.1)*^,^**^,^***
Haemoglobin (g · dl^−1^)	12.8 (1.5)*	9.8 (1.5)*	9.3 (1.5)*^,^**	10.0 (1.1)*	10.5 (1.2)*^,^**
S_a_O_2_ (%)	100 (0)*	100 (0)**	99 (1)***	98 (3)*^,^**^,^***	97 (2)*^,^**^,^***
P_a_O_2_ (mmHg)	354 (128)*	368 (121)**	210 (88)*^,^**^,^***	127 (47) *^,^**^,^***	103 (31) *^,^**^,^***
Arterial pH	7.42 (0.05)*	7.38 (0.05)*^,^**	7.39 (0.08)***	7.33 (0.07)*^,^**^,^***^,^****	7.37 (0.05)*^,^****
Base Deficit	0.3 (2.5)*	−2.0 (3.9)*	−2.0 (2.6)*	−2.3 (2.9)*	−2.3 (2.5)*
S_v_O_2_ (%)	81 (8)*	72 (9)*	71 (8)*	67 (9)*	67 (7)*
Lactate (mmol · L^−1^)	0.58 (0.21)*	1.1 (0.84)*^,^**	1.39 (1.24)*	1.69 (1.61)*^,^**	1.46 (0.77)*
Lactate clearance (%)	38 (24)*	0 (0)*^,†^	−26 (49)*	−55 (112)*^,^**	−51 (74)*^,^**
Norepinephrine (μg kg^−1^ min^−1^)	0.00 (0.00)*	0.03 (0.06)*	0.05 (0.08)*^,^***	0.05 (0.09)*^,^****	0.02 (0.07)***^,^****
Epinephrine (μg kg^−1^ min^−1^)	0.00 (0.00)*	0.01 (0.02)	0.01 (0.03)*	0.01 (0.03)	0.00 (0.01)

*StO*_*2*_, tissue oxygen saturation, *THI* total haemoglobin index, *MAP* mean arterial pressure, *MPAP* mean pulmonary artery pressure, *CVP* central venous pressure, *PCWP* pulmonary capillary wedge pressure, *CI* Cardiac index, *FiO*_*2*_ fraction of inspired oxygen, *S*_*a*_ *O2* arterial oxygen saturation, *P*_*a*_ *O*_*2*_ arterial partial oxygen pressure, *S*_*v*_ *O2* mixed venous oxygen saturation

Data is presented as mean (SD) and *p <0.05* was considered significant between time points: * T −1 vs. T 0-T d1, ** T 0 vs. T1-T d1, *** T 1 vs. T 4-T d1, **** T 4 vs. T d1

**Fig. 1:**
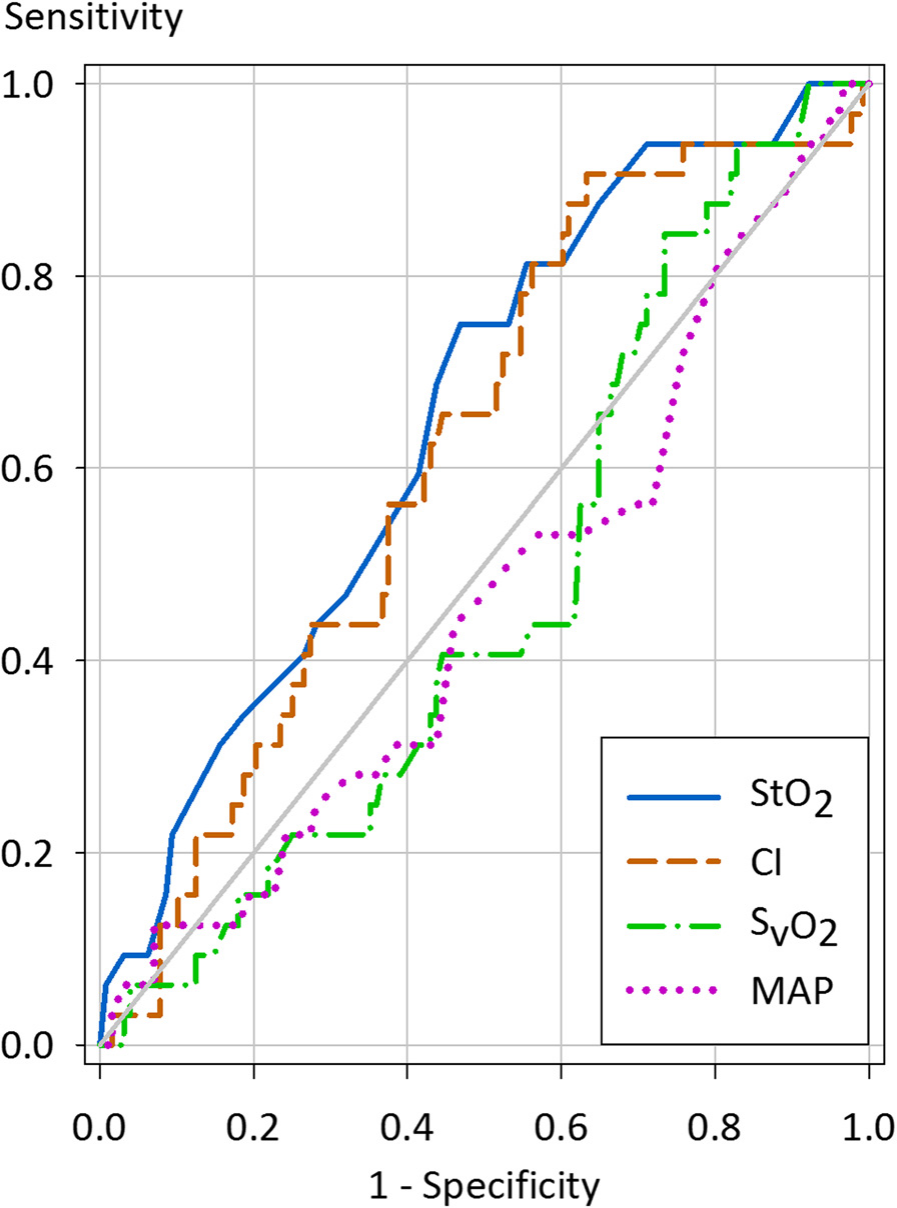
ROC curve of StO_2_ , cardiac index, S_v_O_2_ and mean arterial pressure versus lactate clearance. Measured values from T 0 to T d1 were compared with a lactate clearance of <10 % and ≥10 % at T d1. The area under the curve is 0.65 for StO_2_ (95 % CI 0.55–0.75; *p <*0.01), 0.62 for cardiac index (95 % CI 0.51–0.72; *p <*0.05), 0.47 for S_v_O_2_ (95 % CI 0.37–0.58; *p* = 0.63) and 0.46 for mean arterial pressure (95 % CI 0.35–0.58; *p* = 0.51)

**Fig. 2:**
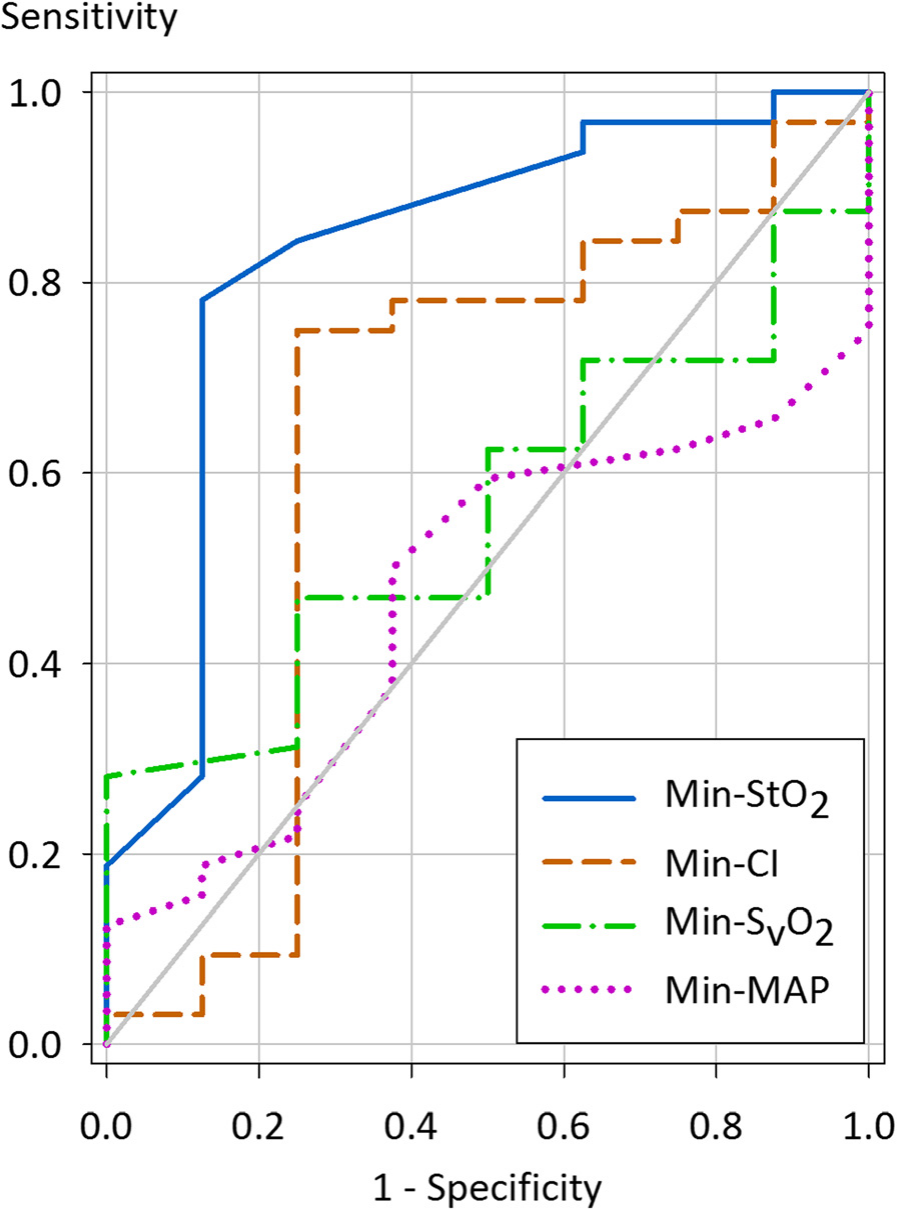
ROC curve of minimum StO_2_, cardiac index, S_v_O_2_ and mean arterial pressure versus lactate clearance. Minimum values of StO_2_ (Min-StO_2_), cardiac index (Min-CI), S_v_O_2_ (Min- S_v_O_2_) and mean arterial pressure (Min-MAP) from T 0 to T d1 were compared with a lactate clearance of <10 % and ≥10 % at T d1. The area under the curve is 0.83 for StO_2_ (95 % CI 0.65–1.02; *p <*0.01), 0.64 for cardiac index (95 % CI 0.37–0.91; *p* = 0.22), 0.56 for S_v_O_2_ (95 % CI 0.36–0.75; *p* = 0.61) and 0.47 for mean arterial pressure (95 % CI 0.27–0.67; *p* = 0.80)

In a ROC-analysis, StO_2_, cardiac index, mean arterial pressure and S_v_O_2_ at the various time points were analysed with respect to their ability to predict a lactate clearance of <10 % at T d1. All these parameters demonstrated an area under the curve (AUC) only close to 0.5 (Fig. 1). An additional ROC-analysis showed that the minimum values of StO_2_, cardiac index, mean arterial pressure and S_v_O_2_ at the pre-defined time points predicted a lactate clearance of <10 % at T d1 with an AUC of 0.83 for StO_2_, 0.64 for cardiac index, 0.47 for mean arterial pressure and 0.56 for S_v_O_2_ (Fig. 2). A minimum StO_2_ *<*75 % was identified as the optimum threshold for a lactate clearance <10 % with a sensitivity of 78 % and a specificity of 88 %. A minimum StO_2_ *<*75 % was observed in 26 patients (65 % of all patients), predominantly at T 1 (n = 17) and T 4 (n = 18) but only two times at T 0 and T d1. Of note, for patients with minimum StO_2_ *<*75 % or ≥75 %, haemodynamic management was not different including therapy with vasopressors, inotropic support, fluid intake and blood transfusion. Analysing minimum StO_2_ and Lactate Clearance at T d1 separately for subgroups with off-pump surgery and surgery with CPB demonstrated no significant differences (Minimum StO_2_ off-pump 71 ± 3 % versus CPB-surgery 74 ± 6 % and lactate clearance at T d1 off-pump −56 ± 68 % versus CPB-surgery −49 ± 76 %).

**Fig. 3:**
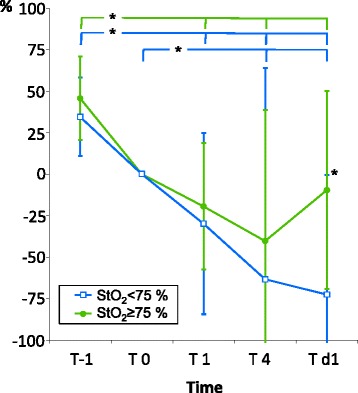
Time course of lactate clearance in subgroups with minimum StO_2_ of at least 75 % or of less than 75 %. For all time points, lactate clearance was calculated referring to T 0 and data are presented as mean with standard deviation. Significance was tested between groups and within groups and a *p <0.05* was considered significant

Univariate and multiple regression analysis showed significant correlations between minimum values of StO_2_ and cardiac index vs. lactate clearance, but not for minimum values of S_v_O_2_ and mean arterial pressure (Table 3). After identification of 13 patients with a maximum lactate concentration higher than 2.0 mmol · l^−1^, univariate analysis of this subgroup demonstrated also a significant correlation of minimum StO_2_ with lactate clearance at T d1 (*r* = 0.63, *p <*0.05). Patients with a minimum StO_2_ ≥75 % demonstrated a significantly better recovery of lactate clearance at day 1 compared to patients with a minimum StO_2_ *<*75 % (Fig. 3). Only the subgroup with a minimum StO_2_ *<*75 % demonstrated a significant increase in creatine kinase MB and troponin T from T 0 to T d1 (Table 4). With respect to the SOFA-scores at T d1, the two subgroups did not differ (Table 5).

**Table 3:** Uni- and multivariate analysis of parameters associated with lactate clearance at day 1

	Univariate analysis				Multivariate analysis			
Factor	Coefficient	95 % Confidence Interval	*r*	*P*	Coefficient	95 % Confidence Interval	*r*	*P*
Minimum StO_2_	0.065	0.024 – 0.105	0.46	<0.01	0.053	0.013 – 0.094	0.46	<0.05
Minimum cardiac index	0.851	0.208 – 1.494	0.40	<0.05	0.637	0.020 – 1.254	0.40	<0.05
Minimum S_v_O_2_	0.014	−0.015 – 0.044	0.16	n. s.				
Minimum mean arterial pressure	−0.004	−0.039 – 0.030	−0.04	n. s.				
Overall							0.55	<0.01

Minimum values of StO_2_, cardiac index, SvO_2_ and mean arterial pressure calculated from values at T0, T1, T4 and Td1

**Table 4:** Marker of muscle and myocardial damage

	n	T 0	T d1	*P*
		After surgery	Day 1	T 0 vs. T D1
Creatine kinase (U L^−1^)				
Overall	40	369 (261)	697 (411)	<0.0001
Min StO2 ≥75 %	14	443 (353)	655 (319)	<0.01
Min StO2 *<*75 %	26	330 (193)	720 (457)	<0.0001
Creatine kinase MB (μg L^−1^)				
Overall	40	27.9 (25.0)	28.4 (19.6)	n. s.
Min StO2 ≥75 %	14	35.3 (36.1)	27.0 (19.1)	n. s.
Min StO2 *<*75 %	26	23.9 (15.8)	29.1 (20.3)	<0.05
Troponin T (μg L^−1^)				
Overall	40	0.80 (1.66)	0.82 (0.95)	n. s.
Min StO2 ≥75 %	14	1.27 (2.73)	1.03 (1.42)	n. s.
Min StO2 *<*75 %	26	0.55 (0.5)	0.71 (0.56)	<0.01

Data is presented as mean (SD) for all patients and for subgroups with minimum StO_2_ of at least 75 % or of less than 75 % at T 0 and T d1

**Table 5:** Intraoperative and outcome data

	Overall	Min StO2 ≥75 %	Min StO2 *<*75 %	*P* between subgroups
Number (n)	40	14	26	
EURO-Score (value)	6 (4)	7 (4)	6 (4)	n. s.
Operation with CPB (n)	33	13	20	n. s.
Duration of surgery (min)	219 (71)	234 (76)	208 (56)	n. s.
Duration of CPB (min)	133 (56)	127 (35)	137 (63)	n. s.
Length of aortic cross clamp (min)	81 (39)	78 (29)	82 (43)	n. s.
SOFA Score T d1 (value)	4 (2)	4 (3)	3 (2)	n. s.
Duration of mechanical ventilation (h)	21 (35)	13 (8)	28 (45)	n. s.
Length of ICU stay (h)	107 (311)	30 (29)	161 (383)	n. s.

Data is presented as mean (SD) for all patients and for subgroups with minimum StO_2_ of at least 75 % or of less than 75 %. Significance was tested between subgroups, but was not significant (n. s.)

## Discussion

In this observational study, near-infrared spectroscopy of peripheral thenar muscle observed impairment and recovery of tissue oxygenation after cardiac surgery. A minimum StO_2_ *<*75 % predicted an impaired lactate clearance much better than cardiac index, mixed venous oxygen saturation or mean arterial pressure. During the observation period markers of myocardial damage increased significantly only in the subgroup with a minimum StO_2_ *<*75 %.

Cardiac surgery with or without cardiopulmonary bypass induces disturbed fluid balance and cardiac output [16, 17], as it was demonstrated in this observational study with reduced S_v_O_2_ after surgery as well as by an increased lactate concentration and base deficit. Clinical studies addressing tissue perfusion and energy metabolism in the perioperative period using microdialysis of the deltoid muscle were contradictory regarding differences between off-pump and on-pump surgery [18, 19]. In our study, a subgroup analysis demonstrated no differences between off-pump and on-pump surgery for tissue oxygenation. Fluid resuscitation as well as positive inotropic and vasoactive drug therapy are recommended to achieve early recovery resulting in sufficient tissue perfusion and oxygen metabolism as main therapeutic goals [1]. Today therapy is managed according to global parameters of haemodynamics and metabolism including cardiac output, S_v_O_2_/S_cv_O_2_, blood pressure, diuresis and lactate concentration [20, 21].

Near-infrared spectroscopy of the thenar muscle, which is monitoring peripheral tissue oxygenation, might allow the more direct observation of tissue perfusion and oxygen delivery after cardiac surgery. A recent observational study using orthogonal polarization spectral imaging for the intermittent qualitative analysis of sublingual microcirculation showed that cardiac surgery is followed by sustained microcirculatory alterations that were correlating with peak lactate levels [22]. Accordingly, in our study StO_2_ values decreased after surgery as an indicator of impaired tissue oxygen saturation with a consecutive increase of lactate concentration and disturbed acid–base balance. Although initial normal StO_2_ values were accompanied by a mean P_a_O_2_ >350 mmHg with a mean FiO_2_ of 0.9–1.0 at T −1 and T 0, a low S_t_O_2_ was also observed together with a high PaO_2_ at T 1 as well as a normal S_t_O_2_ with a PaO_2_ = 103 mmHg at T d1. Ikossi et al. already demonstrated in a prospective trauma study, that S_t_O_2_ did not correlate with PaO_2_ [14]. This data seem to exclude an influence of very high arterial partial oxygen pressure on S_t_O_2_.

Although recovery of StO_2_ occurred at day 1, increased lactate concentration and base deficit persisted probably due to delayed metabolism/wash-out of accumulated acid metabolites. In a recent editorial Vincent described how lactate concentration represents the balance between lactate production and clearance [23]. This is a quite complex process including increased lactate production, lactate release from the cells and transfer of lactate between different tissues and cells as well as diminished clearance [24]. A prospective study with 23 patients demonstrated also a reduced StO_2_ after cardiac surgery, but StO_2_ did not recover until 18–22 h after surgery [9]. Different in house standards for postoperative management may be responsible for the different recovery of tissue oxygenation after 24 h. Continuous measurement of buccal tissue oxygen saturation with a non-invasive, visible-light optical diffusion oximeter could be an alternative method [25]. An actual observational study demonstrated a correlation of buccal StO_2_ and S_cv_O_2_ in 13 children with congenital cyanotic heart disease undergoing a cardiac surgical procedure [26].

Recently, the time course of lactate concentration has been established to monitor global tissue hypoxia during severe sepsis and septic shock and found to be correlated with mortality [15]. Retrospective data suggest, that outcome after cardiac surgery is also correlating with absolute values and trend of lactate concentration [3]. In a prospective observational study with 325 unselected patients early hyperlactatemia immediately after cardiac surgery correlated with morbidity and mortality, probably as a result of intraoperative tissue hypoxia. In contrast, development of hyperlactatemia during ICU stay correlated with epinephrine dose and hyperglycaemia and was inferior with regard to the prediction of outcome [2]. When Pölönen et al. used a goal-oriented algorithm after cardiac surgery to maintain a S_v_O_2_ >70 % and a lactate concentration ≤2.0 mmol l^−1^, morbidity at hospital discharge and length of hospital stay were significantly reduced compared to standard therapy [27].

However, the analysis of the kinetics of lactate production and metabolism precludes real-time observation of sufficient tissue perfusion and requires intermittent blood withdrawals. In contrast, near-infrared spectroscopy is a non-invasive technique that bears the potential to measure directly and continuously the adequacy of tissue oxygenation, as demonstrated in this study. In fact, in our ROC-analysis, a minimum StO_2_ *<*75 % showed the highest predictive value for an impaired lactate clearance at day 1. This threshold is in accordance with the available literature. In trauma patients, a minimum StO_2_ below 75 % correlated with the incidence of postoperative organ dysfunction [6], and mortality of septic shock was associated with a StO_2_ below 78 % after initial resuscitation [7]. Of note, in our study, minimum values of StO_2_ were observed already 1 and 4 h postoperatively. This further highlights the utility of StO_2_ as an early indicator for the later development of a disturbed lactate clearance.

Minimum cardiac output correlated also with lactate clearance and could be an alternative approach, but represents an invasive monitoring technique. Mean arterial pressure and S_v_O_2_, which is routinely approximated by S_cv_O_2_ in clinical practice, did not correlate with lactate clearance and demonstrated the limited value of basic monitoring to estimate lactate clearance. Our study demonstrates that StO_2_ combines the advantage of a non-invasive monitoring technique and the possibility to predict reduced lactate clearance.

Interestingly, only the subgroup of patients with a minimum StO_2_ *<*75 % demonstrated further increasing markers of myocardial injury from ICU admission to day 1. However, this finding was not associated with a deterioration of clinical outcome as assessed by the SOFA-score and length of stay. This is in contrast to another observational study in which decreases in StO_2_ were significantly associated with postoperative morbidity at day 3 and 15 according to the Post-Operative Morbidity Survey [10]. These differences may be attributed to the small number of patients included in our study that prevents reliable information about a possible correlation of minimum StO_2_ and outcome. A limitation of the presented study is the observational design with a limited patient number, but the presented results could be used to develop a goal directed therapy including StO_2_ and test this concept in a prospective clinical trial.

## Conclusions

In the present study, StO_2_ was a non-invasive measurement of tissue oxygenation. A StO_2_ below a critical threshold of 75 % in the early postoperative recovery period was a predictor of a decreased lactate clearance at day 1 after surgery and superior to mean arterial pressure, S_v_O_2_ and even cardiac index. Our data suggest that a StO_2_ below 75 % could be used as an early indicator of persistently increased lactate concentration, which is a recommended marker of tissue perfusion and oxygen metabolism [1]. Further studies are warranted that specifically address the efficacy of a goal-directed therapy based on StO_2_ monitoring to reduce organ dysfunction and morbidity after cardiac surgery.

## Abbreviations

AUC: area under the curve
CI: cardiac index
CVP: central venous pressure
FiO_2_: fraction of inspired oxygen
ICU: Intensive Care Unit
MAP: mean arterial pressure
MPAP: mean pulmonary artery pressure
NIRS: near infrared spectoscropy
PAC: pulmonary artery catheter
P_a_O_2_: arterial partial oxygen pressure
PCWP: pulmonary capillary wedge pressure
ROC: receiver operating characteristic
S_a_O_2_: arterial oxygen saturation
S_cv_O_2_: central venous oxygen saturation
SD: standard deviation
StO_2_: tissue oxygen saturation
S_v_O_2_: mixed venous oxygen saturation
THI: total haemoglobin index
